# Changes in prevalence of nosocomial infection pre- and post-COVID-19 pandemic from a tertiary Hospital in China

**DOI:** 10.1186/s12879-021-06396-x

**Published:** 2021-07-20

**Authors:** Chunmei Su, Zhiqin Zhang, Xu Zhao, Hanlin Peng, Yi Hong, Lili Huang, Jie Huang, Xiangming Yan, Shuiyan Wu, Zhenjiang Bai

**Affiliations:** 1 Department of Nosocomial Infection Control, Children Hospital of Soochow University, Suzhou, Jiangsu China; 2 Intensive Care Unit, Children Hospital of Soochow University, Suzhou, Jiangsu China; 3 Laboratory Department, Children Hospital of Soochow University, Suzhou, Jiangsu China; 4 Department of Cardiology, Children Hospital of Soochow University, Suzhou, Jiangsu China

**Keywords:** Nosocomial infections, Prevalence, COVID-19

## Abstract

**Background:**

Nosocomial infections (NIs) are an important cause of mortality, and increasing evidence reveals that the prevalence of NIs can be reduced through effective prevention and control measures. The aim of this study was to investigate the impact of the prevention and control measures for the COVID-19 pandemic on NIs.

**Methods:**

A retrospective study was conducted to analyze the prevalence of NIs before and after COVID-19 pandemic for 6 months in the Children’s Hospital of Soochow University.

**Results:**

A total of 39,914 patients in 2019 and 34,645 patients in 2020 were admitted to the hospital during the study. There were 1.39% (481/34645) of patients with NIs in 2020, which was significantly lower than the 2.56% (1021/39914) of patients in 2019. The rate of critical and fatal cases was also decreased. In addition, the rate of appropriate handwashing, the number of protective gloves and aprons used per person and the number of healthcare staff per patients were significantly increased. Except for the ICU, the prevalence of nosocomial infection in most departments decreased from 2019 to 2020. Regarding the source of infections, a significant reduction was mainly observed in respiratory (0.99% vs 0.42%, *p* = 0.000) and digestive tract (0.63% vs 0.14%, *p* = 0.000). The microorganism analysis of respiratory infections indicated an obvious decline in acinetobacters and fungi. The most significant decline of pathogens in gastrointestinal infections was observed for rotavirus. The comparison of catheter-related nosocomial infections between 2019 and 2020 did not show significant differences.

**Conclusions:**

The prevention and control measures for the COVID-19 pandemic have reduced the nosocomial infection in almost all departments, except the ICU, mainly regarding respiratory, gastrointestinal, and oral infections, while catheter-related infections did not show any differences.

## Background

Nosocomial infections (NIs) are one of the most common causes of mortality and morbidity in hospitals, affecting hundreds of millions of patients around the world [1, 2]. NIs increase hospital costs through the additional use of drugs and by increasing the length of patients’ hospital stays [3]. Globally, a large number of patients experience NIs, with incidence rates ranging from 3.5 to 12.0% in developed countries and from 5.7 to 19.1% in low-income and middle-income countries [4] In a one-day point prevalence study involving 1265 intensive care units (ICUs) from 76 countries, 51% of patients were found to have NIs [5]. However, the variation reported in the literature can be attributed to the setting, type of hospital, patient population, and the precise definitions and surveillance techniques used in different countries [6]. Evidence shows that the implementation of effective programs regarding NI surveillance can reduce infection rates by approximately one-third [7].

COVID-19 pandemic has changed people’s way of life and raised awareness of infection prevention and control [8, 9]. Additionally, hospitals at all levels have strengthened infection control. However, it is not clear how these preventative initiatives based on the COVID-19 pandemic affect the incidence of nosocomial infections. We conducted this survey to clarify their relationship. To the best of our knowledge, similar data on the pediatric prevalence of nosocomial infections influenced by the COVID-19 pandemic has not been presented previously. The objective of this study was to evaluate the role of the COVID-19 pandemic in the prevalence of nosocomial infections.

## Methods

### Setting

This survey was conducted in the Children’s Hospital of Soochow University, which is a tertiary hospital in Suzhou, China. The hospital is comprised of 23 clinical departments with 1388 beds. The hospital admitted 76,291 inpatients in 2019 and 70,724 in 2020. It also serves more than 2.5 million outpatients every year.

### Confirmation/identification of NIs

Surveys conducted from March 1 to August 31, 2019, were defined as the pre-COVID-19 group, while those conducted from March 1 to August 31, 2020, were defined as the post-COVID-19 group. The age of participants were less than 16 years. Nosocomial infection identification: (1) nosocomial infection warning systems triggered at the laboratory, (2) reports by clinicians, (3) confirmation by staff members of the hospital’s infection-control department, who identified and reported nosocomial infections for which symptoms and signs were present or for which an antimicrobial agent was given. The diagnostic criteria for hospital infections were based on the diagnostic criteria for hospital infection established by the Ministry of Health of the People’s Republic of China [10], which was a modification of the definition from the U.S.’ Centers for Disease Control and Prevention [11].

### Data collection and management

Clinicians retrospectively reviewed medical records to collect basic demographic and clinical data, including information on where the infection came from and what was the most likely pathogen was, before entering data into a web-based data system. The staff of the hospital’s infection-control department reviewed the data from each site for errors and inconsistencies and communicated with clinicians if necessary. Then, clinicians re-reviewed the medical records where necessary to verify the data or make corrections. All methods were carried out in accordance with relevant guidelines and regulations. All experimental protocols were approved by the institutional and/or licensing/ethics committee of Children’s Hospital of Soochow University (2021CS014). Written informed consent was obtained from all subjects and legal guardian/next of kin of deceased patients (dead patients) whose data have been included in the study.

### Statistical analysis

The prevalence of nosocomial infection in different clinical departments were calculated using the number of nosocomial infection patients in each unit out of the total discharged patients from each unit. Patient data were analyzed with the use of SPSS 25.0. The normal distribution measurement data were expressed as mean ± standard deviation (x ± s). The two groups were then compared using the t-test, χ2-test, or exact probability method. The non-normal distribution data were expressed by the quartile method and compared using the Mann–Whitney U test. A *P*-value of less than 0.05 was considered statistically significant.

## Results

### General patient characteristics

From March 1 to August 31, 2019, a total of 39,914 patients were surveyed at the hospital, compared to 34,645 patients surveyed between March 1 and August 31, 2020. The median age of the patients surveyed in 2019 was 4.97 years (IQR:0.92,7.88), while it was 5.39 years (IQR:1.12,8.34) in the 2020 survey group. The incidence rate of nosocomial infection in the post-COVID-19 group was 1.39% (481/34645), which was significantly lower than the 2.56% (1021/39914) in the pre-COVID-19 group. In addition, 3.41% (1361/39914) of patients were critical cases in the pre-COVID-19 group, which was significantly higher than the 2.93% (1014/34654) in the post-COVID-19 group. The rate of appropriate handwashing was 80.69 ± 5.88% in 2020, which was significantly higher than that of 63.73 ± 6.94% in 2019. The number of protective gloves and aprons used per person per month were 101.31 ± 7.10 and 15.42 ± 2.97 before COVID-19, while the number of that were 138.13 ± 17.51 and 34.67 ± 8.62 after COVID-19. The number of healthcare staff per patients after COVID-19 was significantly increased compared to that before COVID-19. Moreover, the mortality rate also declined from 2019 to 2020 (0.13% vs 0.07%, *p* = 0.036). In addition, hospital length of stay were 6.3 days in 2019, whereas that was 5.9 days in 2020(*p* = 0.000) (Table 1).

**Table 1:** The clinical characteristics of patients before and after the emergence of COVID-19

Characteristics	Pre-COVID-19 (*n* = 39,914)	Post-COVID-19 (*n* = 34,645)	*P*
Critical cases	1361 (3.41%)	1014 (2.93%)	0.000
PRISM III of critical cases	11.88 ± 5.87	12.09 ± 6.26	0.232
Number of healthcare staff per patients (ICU)	1.71 ± 0.04	1.84 ± 0.03	0.000
Number of healthcare staff per patients (overall)	1.11 ± 0.04	1.21 ± 0.02	0.000
Rate of appropriate handwashing	63.73 ± 6.94%	80.69 ± 5.88%	0.001
Number of protective gloves used per person per month	101.31 ± 7.10	138.13 ± 17.51	0.001
Number of aprons used per person per month	15.42 ± 2.97	34.67 ± 8.62	0.000
Incidence of NI in critical cases	62 (4.56%)	42 (4.14%)	0.214
Ventilator cases	96 (0.24%)	146 (0.42%)	0.000
Central catheter	113 (0.28%)	85 (0.24%)	0.319
Urinary catheter	665 (1.7%)	623 (1.79%)	0.175
Hospital length of stay (median,days)	6.3	5.9	0.000
Incidence of NI	1021 (2.56%)	481 (1.39%)	0.000
Death	51 (0.13%)	27 (0.07%)	0.036
NI associated death	5 (0.01%)	7 (0.02%)	0.410

*NI* nosocomial infection

### Prevalence of nosocomial infections in different departments of the hospital pre- and post-COVID-19

To reveal the prevalence of nosocomial infections in different units affected by COVID-19, we analyzed their distribution. With the exception of the ICU, the prevalence of nosocomial infection was declining in most departments, including the hematological, gastrointestinal (GI), neonatal, neurological, respiratory, kidney, cardiological, infectious disease, surgery, and endocrine departments. The decline of prevalence was most pronounced in the respiratory and gastrointestinal department. The prevalence was 4.51% (65/1440) pre-COVID-19 compared to 0.99% (12/1216) post-COVID-19 in the GI department. And it was 2.53% (140/5540) pre-COVID-19 compared to 0.51% (20/3956) post-COVID-19 in the respiratory department. The neonatal department and the ICU were the unaccompanied wards. The prevalence of nosocomial infection did not differ significantly between 2019 and 2020 (5.03% vs. 6.20%, *p* = 0.389) in the ICU, but in the neonatal department, the percentages of patients with a nosocomial infection were lower in 2020 than in 2019 (1.75% vs. 3.11%, *p* = 0.007), with a total patient decline due to COVID-19 (Table 2).

**Table 2:** Comparison of prevalence of nosocomial infection among departments

Dept.	Pre- COVID-19	Post- COVID-19	*P*
Hematological	5.29%(316/5975)	3.66%(230/6281)	0.000
ICU	5.03%(33/656)	6.20%(31/500)	0.389
GI	4.51%(65/1440)	0.99%(12/1216)	0.000
Neonatal	3.11%(73/2349)	1.75%(30/1714)	0.007
Neurological	2.82%(80/2832)	1.17%(27/2315)	0.000
Respiratory	2.53%(140/5540)	0.51%(20/3956)	0.000
Kidney	2.53%(53/2099)	1.56%(25/1599)	0.044
Cardiological	2.31%(38/1648)	1.06%(16/1506)	0.007
ID	1.93%(71/3676)	0.57%(12/2112)	0.000
Surgery	1.17%(134/11429)	0.72%(73/10122)	0.001
Endocrine	0.79%(18/2270)	0.15%(5/3324)	0.000

*Dept.* department, *ICU* intensive care unit, *GI* gastrointestinal department, *ID* inflectional disease department

### Types and pathogens of nosocomial infections

**Fig. 1:**
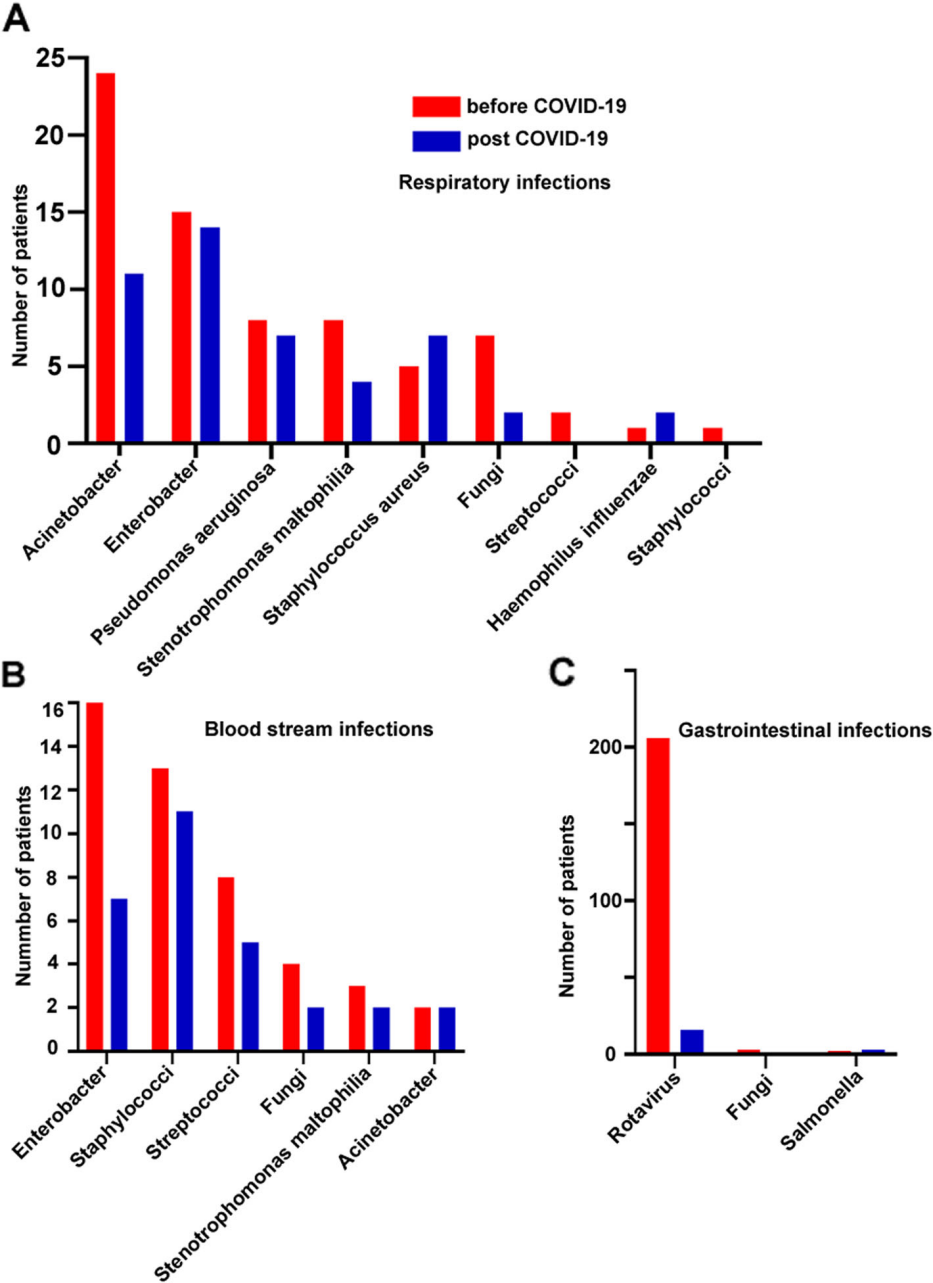
**A** Distribution of pathogens in respiratory-related nosocomial infections; **B** Analysis of isolates of bloodstream infections; **C** Pathogenic microorganism analysis of gastrointestinal infections

The most obvious decline in the post-COVID-19 group was observed in respiratory infections, followed by gastrointestinal infections and oral infections (Table 3). Of 145 respiratory tract infections identified in the 2020 survey, 11 (7.6%) were Acinetobacter infections, and 14 (9.6%) were enterobacterial infections (Fig. 1a).

**Table 3:** Comparison of distribution of nosocomial infections before and after the emergence of COVID-19

Type of infection	Pre-COVID-19 (*n* = 39,914)	Post-COVID-19 (*n* = 34,645)	*P*
Respiratory tract not VAP	0.990%(397/39914)	0.420%(145/34645)	0.000
VAP	0.017%(7/39914)	0.037%(13/34645)	0.097
Gastrointestinal	0.630%(253/39914)	0.140%(48/34645)	0.000
Unknown origin	0.360%(143/39914)	0.260%(91/34645)	0.020
BSI not CLABSI	0.220%(86/39914)	0.260%(91/34645)	0.231
CLABSI	0.020%(8/39914)	0.023%(8/34645)	0.777
Oral	0.180%(73/39914)	0.090%(32/34645)	0.001
UTI	0.070%(28/39914)	0.090%(30/34645)	0.422
CAUTIs	0.015%(6/39914)	0.032%(11/34645)	0.132
Surgical site	0.070%(26/39914)	0.070%(25/34645)	0.715

*VAP* ventilator-associated pneumonia; unknown origin refers to infection with unknown pathogen and infection site, *CLABSIs* central line-associated bloodstream infections, *CAUTIs* catheter-associated urinary tract infections, *UTI* urinary tract infection

The leading pathogen of gastrointestinal nosocomial infections was rotavirus, followed by fungi, and these two pathogens were also the ones that showed the most significant decline in COVID-19 exposure (Fig. 1b). Most of the oral nosocomial infections we describe here were thrush, which was significantly reduced after the emergence of COVID-19. Although the percentages of patients with central line-associated blood stream infections (CLABSIs) did not differ significantly between 2019 and 2020 (Table 4), the percentages of Enterobacteriaceae in patients with CLABSIs were lower in 2020 than in 2019 (Fig. 1c).

**Table 4:** Analysis of catheter-associated nosocomial infections before and after the emergence of COVID-19

Catheter sites	Pre-COVID-19 (*n* = 987)	Post-COVID-19 (*n* = 941)	*P*
VAP	7.29%(7/96)	8.90%(13/146)	0.656
CAUTIs	0.90%(6/665)	1.77%(11/623)	0.181
CLABSIs	3.54%(8/226)	4.65%(8/172)	0.576

*VAP* ventilator-associated pneumonia, *CLABSIs* central line-associated bloodstream infections, *CAUTIs*, catheter-associated urinary tract infections

### Catheter-associated nosocomial infections

In 2019, catheters were used in 987 patients, 21 (2.13%) of whom developed catheter-related infections, compared with 941 patients who used catheters and 32 (3.40%) who developed catheter-related infections in 2020. These patients were mostly in the ICU. Pre- and post-COVID-19, the highest prevalence of catheter-associated nosocomial infections was ventilator-associated pneumonia (VAP), followed by CLABSIs and catheter-associated urinary tract infections (CAUTIs). There was no statistically significant difference in nosocomial infections between the three catheter-associated nosocomial infections pre- and post-COVID-19 emergence.

## Discussion

Nosocomial infections are one of the most common complications affecting hospital patients and greatly increase morbidity and mortality, often resulting in a prolonged hospital stay. Preventing nosocomial infections, therefore, presents an important challenge to clinicians and health service managers [1–3]. In this point-prevalence survey conducted in our center, we found that nosocomial infections affected 1.39% of hospitalized patients in 2020—a significantly lower percentage than we observed in a survey conducted in 2019, which was 2.56%. The prevalence was lower than that stated in previous reports from other Chinese cities and other countries [4, 12, 13]. The overall mortality rate of the point survey in 2019 was 0.13%, while the mortality in 2020 was 0.07%, which may have been due to the reduction of nosocomial infection prevalence. Nosocomial infection was a main factor causing mortality of patients in hospitals. The median length of stay of inpatients was 5.9 days, which is shorter than that of patients with 6.3 days before COVID-19. Moreover, the rate of appropriate handwashing, the number of protective gloves and aprons used per person and the number of healthcare staff per patients after COVID-19 were significantly increased compared to that before COVID-19. Our data first indicated that the significant reductions in the prevalence of nosocomial infections that were achieved in our hospital were due to preventative initiatives based on COVID-19 controlling.

With the emergence of the COVID-19 pandemic, several resources with recommendations for the prevention of COVID-19 transmission have been developed. A series of recommendations based on strategies outlined in the 2019 guidelines and the current background and epidemic situation for the management of COVID-19 were released in 2020 [9]. In addition, the guidelines provide regional prevention strategies for local health department implementation. Our hospital has also developed region-specific resources and tools to guide facilities in their COVID-19 prevention efforts.

Our results provide evidence of success in preventing nosocomial infections. The data showed that the prevalence of nosocomial infection declined in most departments, including the hematological, gastrointestinal (GI), neonatal, neurological, respiratory, kidney, cardiological, infectious disease (ID), surgery, and endocrine departments, excluding the ICU. The neonatal department and the ICU were the unaccompanied wards with strict isolation and aseptic operation in our hospital, which are also relevant prevention measures during the COVID-19 pandemic. The prevalence of nosocomial infection did not differ significantly between 2019 and 2020 in the ICU, but for the neonatal department, the percentages of patients with a nosocomial infection were lower in 2020 than they were in 2019, with a total patient decline due to COVID-19. Thus, inadequate resources are still a problem that we need to confront in our center.

The decline of prevalence was most pronounced in respiratory and digestive tract infections. Respiratory infections were the most common nosocomial infections with a prevalence of 38.9%, while the rate was decreased to 30.1% with changes after COVID-19 emerged. COVID-19 spreads by droplets shed of the respiratory system by someone infected with the virus, which means it would spread faster with higher proximity of people, larger contact networks, and lower levels of hygiene. During the COVID-19 epidemic, masks, gloves, and hand sanitizer were used to prevent the transmission owing to the fact that respiratory droplets and contact transmission are the main routes of transmission of this disease [9]. This is why the prevalence of respiratory nosocomial infection was reduced significantly. Pathogen analysis of patients with respiratory tract infection showed that gram-negative bacteria, such as Acinetobacter, Enterobacter pylori, *Pseudomonas aeruginosa*, *Staphylococcus aureus*, and fungi, were the main pathogens causing nosocomial infections. Acinetobacter baumannii is an opportunistic human pathogen that predominantly infects critically ill patients. In contrast to a previous report [14], our data indicate that Acinetobacter infection rates are higher compared to other gram-negative pathogens. In light of this, continual public health monitoring and prevention activities are needed aside from the measures taken during the COVID-19 pandemic.

In pediatrics, rotavirus is the main pathogen of gastrointestinal infection, and under the coronavirus prevention measures, the rotavirus is also one of the pathogens with the most obvious decline in nosocomial infection. Rotavirus is considered to be a major cause of infant and childhood morbidity and mortality, particularly in developing countries [15]. Thus, it is vital to monitor its prevalence. These results indicate that the preventive measures implemented during the COVID-19 pandemic can significantly reduce the incidence of rotavirus infection. Therefore, we believe that relevant measures should be maintained in future work.

Although the percentages of patients with CLABSIs did not differ significantly between 2019 and 2020, the percentages of Enterobacteriaceae in patients with central line-associated bloodstream infections were lower in 2020 than in 2019. As we all know, the recommended approach to prevent Enterobacteriaceae transmission, which was enhanced during the COVID-19 pandemic, is improved hand hygiene [16, 17]. Besides, CAUTIs mostly occurred in the critical care unit, and there was no significant reduction in catheter-related infection. In addition to the standard and transmission-based precautions for critically ill patients, several strategies focused on the prevention of specific nosocomial infections, such as ventilator-associated pneumonia (VAP), CLABSIs, and CAUTIs, are needed [18–20]. To reduce the incidence rate of catheter-related infection for critically ill patients, more evidence-based interventions should be boosted, in addition to the preventative measures based on the COVID-19 pandemic.

We acknowledge that there are some limitations to this study. First, since the study was based on surveillance data, we did not have information on the patients’ specific underlying diseases and the severity of their medical conditions, which has certainly influenced the patients’ outcomes. Second, we did not have data on whether the antimicrobial therapy was appropriate or not, nor data on delays in the commencement of the treatment.

## Conclusions

The prevention and control measures for the COVID-19 pandemic have reduced the nosocomial infection rate in almost all departments, except the ICU, mainly in respiratory, gastrointestinal, and oral infections, while bloodstream infections and catheter-related infections did not show any difference before and after the COVID-19 pandemic.

## Data Availability

The datasets used and/or analysed during the current study are available from the corresponding author on reasonable request.

## Abbreviations

NI: Nosocomial infection
IQR: Interquartile range
ICU: Intensive care unit
GI: Gastrointestinal department
VAP: Ventilator-associated pneumonia
CLABSIs: Central line-associated blood stream infections
CAUTIs: Catheter-associated urinary tract infections
UTI: Urinary tract Infection
